# Transcription Elongation Factor GreA Has Functional Chaperone Activity

**DOI:** 10.1371/journal.pone.0047521

**Published:** 2012-12-12

**Authors:** Kun Li, Tianyi Jiang, Bo Yu, Limin Wang, Chao Gao, Cuiqing Ma, Ping Xu, Yanhe Ma

**Affiliations:** 1 National Engineering Laboratory for Industrial Enzymes, Institute of Microbiology, Chinese Academy of Science, Beijing, People's Republic of China; 2 State Key Laboratory of Microbial Metabolism & School of Life Sciences and Biotechnology, Shanghai Jiao Tong University, Shanghai, People's Republic of China; 3 State Key Laboratory of Microbial Technology, Shandong University, Jinan, Shandong Province, People's Republic of China; 4 University of Chinese Academy of Sciences, Beijing, People's Republic of China; Arizona State University, United States of America

## Abstract

**Background:**

Bacterial GreA is an indispensable factor in the RNA polymerase elongation complex. It plays multiple roles in transcriptional elongation, and may be implicated in resistance to various stresses.

**Methodology/Principal Findings:**

In this study, we show that *Escherichia coli* GreA inhibits aggregation of several substrate proteins under heat shock condition. GreA can also effectively promote the refolding of denatured proteins. These facts reveal that GreA has chaperone activity. Distinct from many molecular chaperones, GreA does not form stable complexes with unfolded substrates. GreA overexpression confers the host cells with enhanced resistance to heat shock and oxidative stress. Moreover, GreA expression in the *greA/greB* double mutant could suppress the temperature-sensitive phenotype, and dramatically alleviate the *in vivo* protein aggregation. The results suggest that bacterial GreA may act as chaperone *in vivo*.

**Conclusions/Significance:**

These results suggest that GreA, in addition to its function as a transcription factor, is involved in protection of cellular proteins against aggregation.

## Introduction

RNA synthesis is a conserved biochemical reaction mediated by DNA-dependent RNA polymerase (RNAP) in all organisms. In the 3 steps of transcription—initiation, elongation, and termination—a host of transcription factors interact with RNAP and regulate its enzymatic activity. Transcription elongation factor GreA, also named as transcription cleavage factor, is one of the conserved factors in nascent mRNA synthesis [1]–[3].

GreA was first reported as a 158 amino acid product of the *greA* gene that can suppress the temperature-sensitive mutation in the RNA polymerase β subunit [1]. Borukhov *et al*. demonstrated that GreA can induce cleavage and removal of 3′-proximal dinucleotides from the nascent RNA, which allows the newly generated 3′-terminus to be extended into longer transcripts. This step appears to allow the transcriptional ternary complex to resume transcription from the indefinite elongation arrest often induced by a specific DNA site [4]. GreA was also reported to cleave transcripts containing misincorporated residues preferentially in the inactivated state of elongation, which increases transcription fidelity and may also prevent formation of “dead-ends” *in vivo* [2]. Besides, GreA and its homolog, GreB, are also involved in the transition from transcription initiation to elongation [5], as they may facilitate the escape of the RNAP complex from certain promoters. Both proteins have also been reported to act as transient catalytic components of RNA polymerase [6].

The crystal structures of GreA in *Escherichia coli* [7] and its paralog Gfh1 in *Thermus aquaticus* [8] have an overall “L-shaped” structure composed of a C-terminal domain (CTD) and an N-terminal domain (NTD). Interestingly, the “L-shaped” structure is also shared by some proteins with molecular chaperone activity, such as FimC [9], PapD [10], HscB [11], and SfaE [12]. In these proteins, 2 immunoglobulin-like domains are located at an approximate 90° to each other with a large cleft between them. This structural trait may contribute to their chaperone functions. Furthermore, GreA was reported to be involved in acid, salt, and cold stress responses in *Streptococcus mutans* [13], *Sinorhizobium meliloti* [14], *Rhizobium tropici* [15], and *Shewanella livingstonensis* [16]. A *greA* and *greB* double-mutant strain is not able to survive at high temperatures [17]. Together, these data indicate that GreA may play important roles in stress resistance, in addition to its role in transcription elongation.

Living organisms are often exposed to various environmental stresses such as extreme pH, non-optimal temperatures, osmotic pressures, and free radicals, which cause inactivation and aggregation of cellular proteins and threaten organismal survival. Organisms have evolved complicated systems to resist these potential stresses. Molecular chaperones are of particular significance in these systems. Molecular chaperones belong to a large family of proteins that prevent protein aggregation and/or promote refolding of non-native proteins. They are ubiquitous in all 3 kingdoms, and many of them are highly conserved [18]. Except for typical chaperones like DnaK, GroEL, and small heat shock proteins (sHSPs), many proteins with well-characterized non-chaperone activities have now been shown to possess chaperone activity. Examples include EF-G [19], EF-Tu [20], thioredoxin [21], protein disulphide isomerase (PDI) [22], and ribosomes [23]. These observations suggest that intracellular chaperones are widely distributed among diverse functional classes.

In this study, we show that *E. coli* GreA has chaperone activity, as evidenced by both repression of aggregation and reactivation of denatured proteins. When overexpressed, GreA enhances the resistance of host cells to environmental perturbations. GreA-expression also suppresses temperature-sensitive phenotype of *greA/greB* double mutant by alleviating cellular aggregation. To the best of our knowledge, this may be the first report of a transcription factor that has chaperone function. We therefore propose that in addition to its function as a transcription factor, GreA may play a role in protein quality control *in vivo*.

## Results

### GreA prevents heat-induced aggregation

**Figure 1:**
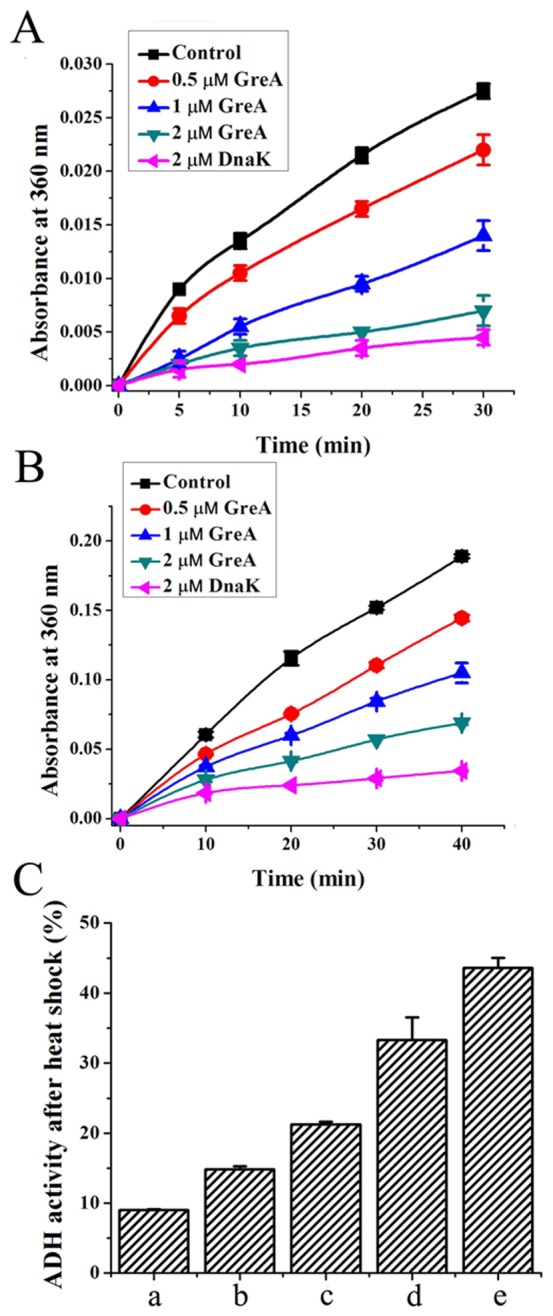
GreA inhibits heat-induced aggregation of substrate proteins. (A) ADH aggregation at 48°C is suppressed in the presence of GreA. The control, 0.2 µM, 0.5 µM, 1 µM, 2 µM GreA or 2 µM DnaK was added to 1 µM ADH. Aggregation was started by incubation at 48°C and detected by optical density at 360 nm. (B) GreA inhibits aldolase aggregation at 50°C. 1 µM aldolase containing 0.5 µM, 1 µM, 2 µM GreA or 2 µM DnaK was incubated at 50°C. Aldolase only was set as a control. The aggregation was detected by optical density at 360 nm. (C) GreA protects ADH enzymatic activity under heat shock conditions. 0.3 µM ADH with 0.3 µM (b), 0.6 µM (c), 1.2 µM GreA (d), 1 µM DnaK (e) or ADH only (a) was incubated at 50°C. The enzymatic activity was measured after incubation for 80 min.

Most molecular chaperones inhibit the stress-induced aggregation of various client proteins nonspecifically. In order to test whether GreA could act in a similar manner as other molecular chaperones, we tested its anti-aggregation activity. Alcohol dehydrogenase (ADH) was used to study the influence of GreA on protein aggregation. As shown in Figure 1A, when incubated at 48°C, ADH rapidly formed insoluble aggregates in the absence of GreA at this temperature, indicated by a dramatic increase in optical turbidity. However, when this experiment was performed in the presence of GreA, the aggregation was obviously alleviated. These results suggest that during thermal stress, GreA protects unfolded proteins from irreversible aggregation.

We also observed that GreA also exhibited chaperone activity towards aldolase. As shown in Figure 1B, aldolase was denatured and aggregated at 50°C. The thermally induced aggregation of 1 µM aldolase was partially inhibited by 0.5 µM GreA. When the concentration of GreA was increased to 2 µM, the aggregation of aldolase was largely suppressed. These results suggest that the anti-aggregation activity of GreA is not restricted to specific substrates, and that the protein has general chaperone activity.

### GreA protects enzymatic activities against heat shock

Enzymatic activity is a sensitive measure of the native or denatured state of proteins. As heat-induced denaturation and aggregation occurs, it is accompanied by a loss of enzymatic activity. Here, we used ADH to test the protective effect of GreA on enzymatic activity. As indicated in Figure 1C, after incubation for 80 min at 50°C, ADH lost about 90% activity in the absence of GreA. However, when GreA and ADH were co-incubated at a molar ratio of 4∶1, ADH retained more than 30% activity, indicating that GreA can also preserve enzyme activity during thermal stress.

### GreA promotes refolding of denatured proteins

**Figure 2:**
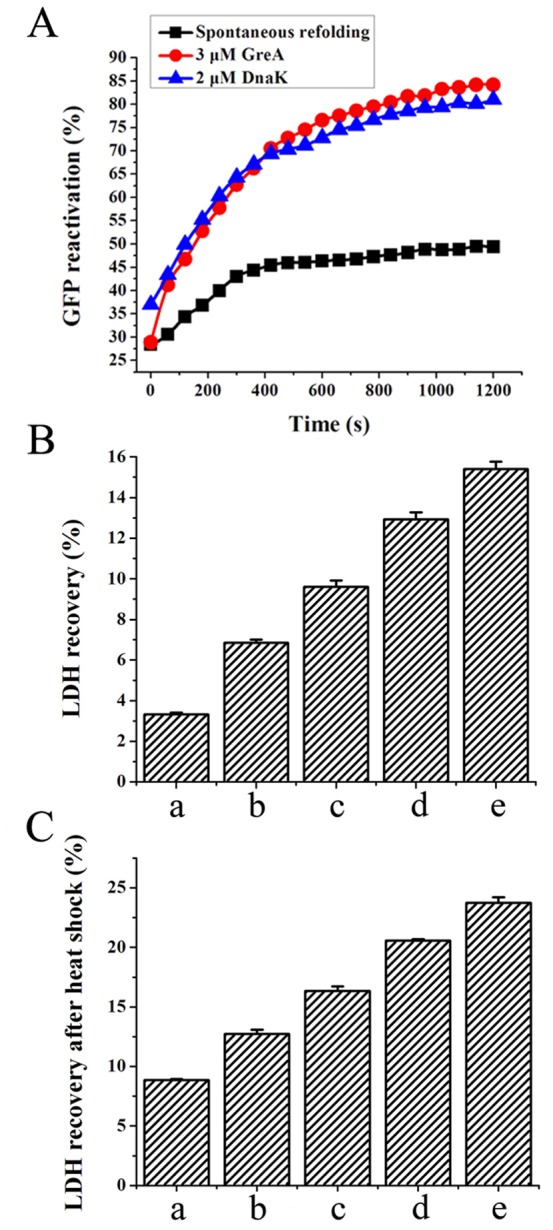
GreA facilitates protein reactivation from unfolded state. (A) GreA facilitates GFP refolding. GFP (100 µM) was denatured in 0.12 M HCl for 60 min and then diluted 100-fold. Spontaneous refolding or in the presence of 3 µM GreA or 2 µM DnaK was monitored using a Fluostar Optima microplate reader. (B) GreA promotes LDH refolding after GnHCl denaturation. LDH (15 µM) denatured by 6 M GnHCl was diluted 100-fold to start spontaneous refolding or GreA-facilitated refolding. (a) Control (b) 0.3 µM GreA (c) 0.6 µM GreA (d) 1.2 µM GreA (e) 1.2 µM DnaK. (C) GreA promotes LDH refolding after heat denaturation. 0.2 µM LDH was incubated at 50°C for 80 min. After cooling down, 0.2 µM, 0.4 µM, 0.8 µM GreA or 0.5 µM DnaK was added to start refolding and the final concentration of LDH was adjusted to 0.1 µM. The enzymatic activity was detected after recovery for 30 min. (a) Control (b) 0.2 µM GreA (c) 0.4 µM GreA (d) 0.8 µM GreA (e) 0.5 µM DnaK.

Most molecular chaperones can promote protein refolding in addition to preventing protein aggregation [24]. We therefore used 3 proteins to assess the ability of GreA to promote protein refolding. Correct folding was investigated by measuring the biological activities after co-incubation with GreA in refolding buffer. As shown in Figure 2A, in the absence of GreA, HCl-denatured green fluorescent protein (GFP) spontaneously refolded after 100-fold dilution. Ultimately, a refolding percentage of 50% was achieved. Addition of 3 µM GreA dramatically increase the refolding percentage to 84%.

Lactate dehydrogenase (LDH) was used as another substrate. As shown in Figure 2B, after 100-fold dilution and incubation for 30 min, guanidine hydrochloride (GnHCl)-denatured LDH spontaneously refolded to 3.3% of the original enzymatic activity. In the presence of 0.3 µM GreA, the refolding percentage was elevated to nearly 7%. When the GreA protein was added to 1.2 µM, the LDH refolding percentage increased by more than 3-fold. Heat-denatured LDH could also be refolded in the presence of GreA (Figure 2C). Together, these results show that GreA can promote the refolding of denatured proteins, protect them from aggregation, and therefore preserve their enzymatic activity.

### GreA does not form complexes with denatured substrates

**Figure 3:**
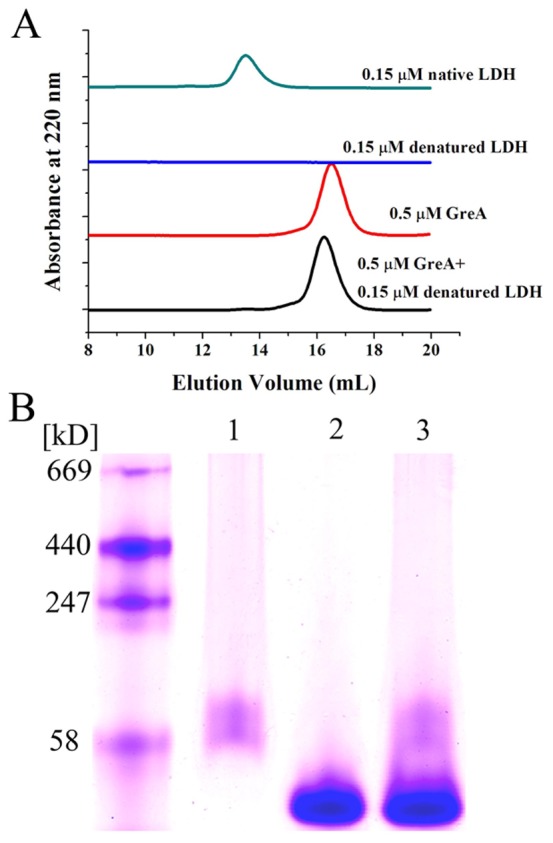
GreA does not bind to the denatured substrate. (A) Denatured LDH cannot bind to GreA, as indicated by SEC. LDH (15 µM) was denatured in 6 M GnHCl and then diluted 100-fold in the presence or absence of 0.5 µM GreA. The change in the molecular size was detected using SEC. As control, 0.15 µM native LDH or 0.5 µM GreA was both loaded onto SEC. (B) Gradient native electrophoresis showed that denatured ADH did not bind to GreA. (1) GnHCl denatured ADH (2) GreA (3) Co-incubated GreA and denatured ADH.

Many molecular chaperones can preferentially recognize and bind to denatured proteins to form complexes. This binding capacity is closely related to their anti-aggregation activity [24]. Here, we used size exclusion chromatography (SEC) and non-denaturing gradient gel electrophoresis to detect the interaction of GreA with denatured proteins. As shown by SEC (Figure 3A), after incubation in GnHCl, LDH was mostly denatured and no elution peak could be observed. However, the GreA elution peak showed little change whether the GreA sample was co-incubated with denatured LDH or not. To further elucidate its binding property, we used ADH as another substrate. As indicated in Figure 3B, after co-incubation with GnHCl-denatured ADH, neither the GreA lane nor the ADH lane showed any change. Furthermore, no complex was detected. We propose that distinct from most molecular chaperones, GreA does not bind to denatured substrates and form complexes, indicating that alternative mechanisms are responsible for its chaperone function.

### Hydrophobicity of protein GreA

**Figure 4:**
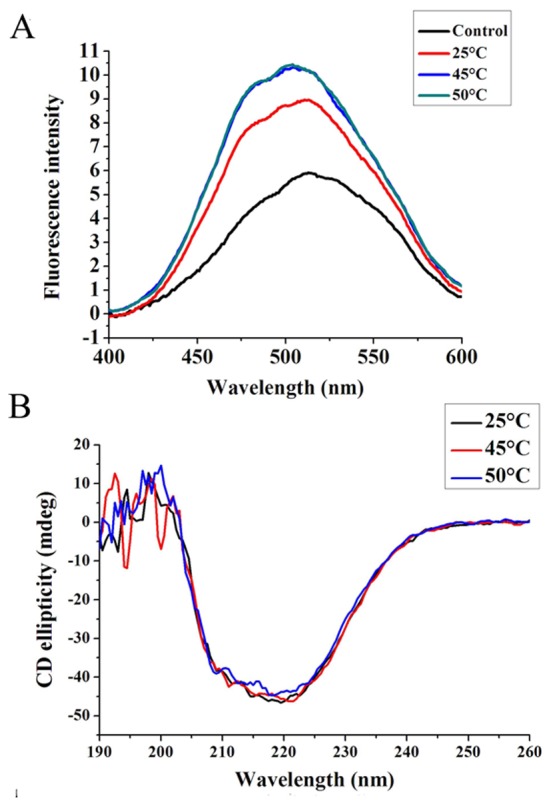
Primary and secondary structure analysis of GreA. (A) The ANS binding experiment shows the hydrophobicity of GreA under normal or heat-shock conditions. ANS alone was set as control; 2 µM GreA was incubated at 25°C, 45°C, or 50°C for 60 min and then mixed with 40 µM ANS. Fluorescence spectra were recorded after incubation for 20 min. (B) CD shows that the secondary structure change of GreA during heat shock was very subtle. The CD ellipticity of GreA was scanned after incubation at 25°C, 45°C, or 50°C for 60 min.

Both the hydrophobicity and hydrophilicity of the GreA molecule have been demonstrated by crystal structure analysis. A binding experiment using 8-anilino-1-naphthalene sulfonic acid (ANS) also underscored the hydrophobic nature of GreA (Figure 4A). As the temperature increased, more ANS molecules became bound to the GreA molecule, resulting in increased fluorescence intensity. This indicated that more hydrophobic domains were exposed as the temperature rose. However, the circular dichroism (CD) results suggested that the structural change in this process is minimal (Figure 4B). As indicated by the CDNN analysis, only subtle changes in the secondary structure were detected (Table 1).

**Table 1:** CDNN analysis of the CD data.

	25°C	45°C	50°C
Helix	99.3%	99.3%	99.3%
Anti-parallel	0.0%	0.0%	0.0%
Parallel	0.1%	0.1%	0.2%
Beta-turn	3.9%	3.8%	3.9%
Radom coil	0.2%	0.2%	0.2%
Total sum	103.6%	103.5%	103.5%

### GreA overexpression enhances bacterial stress resistance

**Figure 5:**
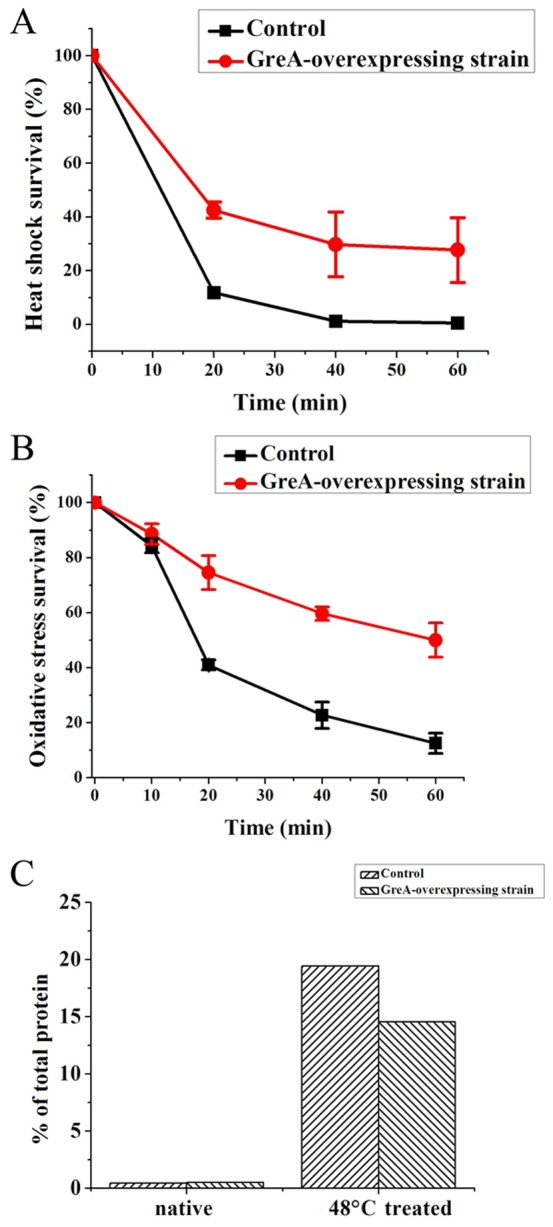
Enhanced resistance of the GreA-overexpressing strain to environmental stresses. (A) GreA overexpression results in enhanced heat shock survival. The IPTG-induced GreA-overexpressing strain and the control strain were both challenged by heat shock at 48°C. The viability of cells was estimated by counting the number of surviving cells on the plates. (B) GreA overexpression confers the host strain with enhanced resistance to oxidative stress. The GreA-overexpressing strain and the control strain were both challenged with 5 mM H_2_O_2_ after induction with IPTG for 1 h. Surviving cells on the plates were calculated to estimate the survival rate. (C) GreA expression suppresses cellular aggregation. The IPTG-induced strains were heat shocked at 48°C for 0 min or 40 min, and the cellular aggregates were isolated and qualified.

To further determine the physiological functions of GreA *in vivo*, we tested the effect of GreA-overexpression on cellular resistance to environmental stresses. As reported earlier, overexpression of certain chaperones can protect cellular proteins from aggregation, which endows the host cell with stress resistance [25]–[28]. Herein, we used the GreA-overexpressing *E. coli* BL21 (DE3) strain to validate the effect of GreA on resistance to high temperature and oxidizing conditions. The strain containing an empty vector was used as the control. In the heat shock experiment, both strains were challenged by treatment at 48°C for various time-periods after isopropyl-b-d-1-thiogalactopyranoside (IPTG) induction for 1 h. As shown in Figure 5A, after 60 min, the GreA-overexpressing strain had a survival rate of 27.7%. In contrast, almost no survival was observed for the control strain. To confirm that the enhanced resistance is due to the chaperone function of GreA, the cellular aggregates after heat shock have also been quantified. As shown in Figure 5C, the control strain showed more extensive aggregation than its counterpart strain. These results suggest that the presence of excess GreA molecules may prevent the heat-induced loss of cell viability by its chaperone function.

To assess the oxidative resistance of GreA-overexpressing strain, we challenged cells with 5 mM H_2_O_2_ and tested the survival rate. Following a 60-min challenge, the GreA-overexpressing strain had a survival rate of about 50%, while the control strain showed a survival rate of 12.5% (as shown in Figure 5B). Together, these results demonstrate that GreA overexpression confers the host cells with enhanced resistance to various environmental stresses.

### Effect of GreA-expression on *greA/greB* double mutant strain

**Figure 6:**
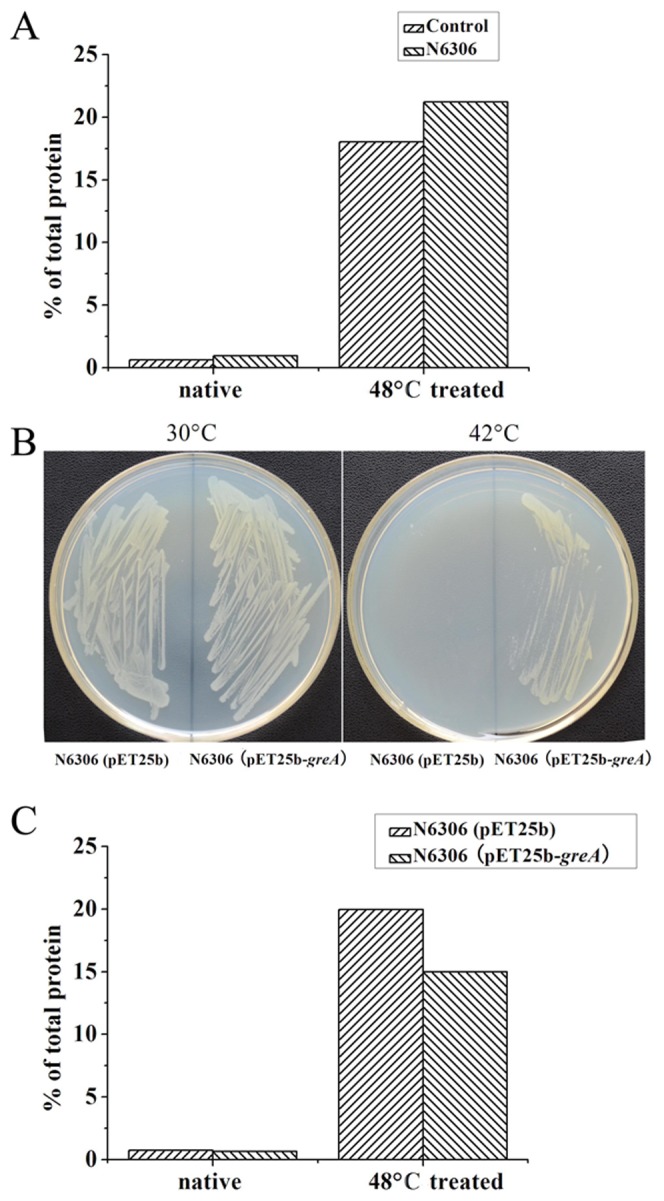
Effect of *greA/greB* double mutation on cellular aggregation. (A) The *greA/greB* double mutant strain N6306 suffers extensive aggregation under heat shock. (B) GreA-expression in N6306 suppresses the temperature-sensitive phenotype. (C) GreA-expression in N6306 alleviates cellular aggregation under heat shock.

The *greA/greB* double mutant strain has been reported temperature sensitive [29]. Although the sensitivity may relate to the transcriptional function of GreA, we propose this phenotype may also result from deficiency of GreA as *in vivo* chaperone. To confirm this hypothesis, we isolated and qualified the aggregated proteins from the *greA/greB* double mutant strain N6306 under heat shock. As shown in Figure 6A, the cellular aggregation of N6306 strain is more severe than the control strain, indicating that the cellular proteins are more vulnerable to misfolding/aggregation in the absence of GreA/GreB. This result also suggests that GreA may play chaperone function *in vivo*. Unsurprisingly, when GreA was expressed in the double mutant, the temperature-sensitive phenotype was obviously suppressed. As shown in Figure 6B, the GreA-expressing strain can grow at the temperature as high as 42°C while the control cannot. We also cultured the GreA-expressing N6306 strain and qualified the aggregates after 48°C heat shock as described above. As shown in Figure 6C, GreA expression in the *greA/greB* double mutant dramatically alleviates the *in vivo* protein aggregation. These results above suggest that GreA may act as chaperone *in vivo*.

## Discussion

GreA, a well-studied transcription factor in prokaryotes, has been reported to participate in several transcription-related processes [2]–[6]. However, there is little evidence to suggest that transcription factors also have chaperone properties. Here, we show that the transcriptional elongation factor GreA suppresses protein aggregation and promotes reactivation of denatured proteins, which provide evidence that GreA also has chaperone activity. Although the activity of GreA is not so effective as DnaK, concerning the small molecular size (about 17 kD) and its main function as a transcription factor, we propose that the chaperone activity is notable.

The crystal structure of GreA from *E. coli*, determined in 1995, revealed that the surface of the molecule is negatively charged uniformly, while the opposite surface has some hydrophobic patches. The ANS-binding experiment also confirmed the hydrophobic nature of the GreA protein. It has been previously suggested that GreA hydrophobic patches may act as a binding surface for interaction with the RNA polymerase complex. However, the hydrophobic patch also serves as a characteristic for some chaperone proteins [30]–[32], and we therefore reasoned that GreA may also utilize the hydrophobic patches for chaperone-like activity. Interestingly, as temperature was elevated, the hydrophobicity of GreA moderately increased while the secondary structure changed very slightly. Generally, the heat-induced hydrophobicity increase is usually associated with protein unfolding. However, at the relatively high temperature of 50°C, GreA maintains its ability to suppress the aggregation of substrate proteins. We propose that because of the high hydrophilicity of GreA (negatively charged surface), a slight increase in the hydrophobicity may not be severe enough to result in GreA unfolding, rather, it may provide more interacting surfaces for interaction with client proteins.

Distinct from many other molecular chaperones, the binding capacity of GreA to the denatured substrates is very weak. Indeed, no obvious chaperone-substrate complexes were detected. This characteristic is similar to that of *E. coli* proteins of the thioredoxin family, namely, Trx, and YbbN [33], which have been reported to promote the refolding of denatured substrates but do not preferentially bind unfolded proteins. Although little sequence homology was observed between GreA and thioredoxins, we propose that they may share a similar mechanism regarding their chaperone functions.

There are approximately 13,800 molecules of GreA in each *Bacillus subtilis* cell, which is nearly twice that of RNAP levels and far more than that of other transcription factors [34]. The distribution of highly concentrated GreA molecules in the cell may engender an effective chaperone buffer like DnaK and other chaperones. In turn, this would help to prevent protein aggregation, promote renaturation of denatured proteins, and thus enhance cellular resistance to stress. Our result that the temperature sensitive *greA/greB* double mutant strain suffers more extensive protein aggregation suggests that GreA may act as chaperone *in vivo*. Increased expression of GreA under acidic stress [13] and the enhanced heat-shock survival rate of the GreA-overexpressing strain provide extra evidence for such activity.

Deletion of *greA* results in sensitivity to salt stress [14], [15] and double-deletion of *greA* and *greB* causes heat sensitivity [17], which suggest that GreA plays a critical role in stress resistance. Owing to the chaperone activity of GreA, we infer that GreA may protect or stabilize RNAP in stressful conditions. If this is one of the major roles of GreA, we predict that RNAP should be one of its natural substrates. We further propose that GreA may play a novel role in the transcription apparatus. Interestingly, the Database of Interaction Protein (DIP) (http://dip.doe-mbi.ucla.edu/dip/Main.cgi) shows that GreA interacts directly with ribosome subunits, such as DnaK, DnaJ, GroES, ClpX, and other chaperones *in vivo*, suggesting the existence of potentially important relationships between GreA and the molecular chaperone system. In conclusion, this study may provide the first evidence that indicates a link between the transcription apparatus and protein quality control.

## Experimental Procedures

### Construction, expression, and purification of recombinant GreA

We amplified the *greA* and *dnaK* gene from *E. coli* genomic DNA (from strain DH10B) by PCR. The product was digested with NdeI and HindIII, and then ligated to pET28a (Novagen) expression vector. The GFP gene from the jellyfish *Aequorea victoria* was ligated to pET28a vector via the SacI and NheI cohesive termini. The ligated plasmids were transformed into *E. coli* BL21 (DE3) strain and sequences were confirmed by DNA sequencing.

The overexpressing strain was cultured to OD_600_ = 0.4 and induced with 1 mM IPTG at 37°C for 5 h. Cells were harvested and washed once with phosphate buffer (pH 7.4). The pellet was resuspended with binding buffer (20 mM sodium phosphate, 0.5 M NaCl, 20 mM imidazole, pH 7.4) with 10% glycerol, 1 mM DTT, and 1 mM phenylmethanesulfonyl fluoride (PMSF), and lysed by sonication. After centrifugation at 12, 000 rpm for 10 min, the supernatant was loaded on a 5-mL HisTrap column and eluted with the elution buffer (20 mM sodium phosphate, 0.5 M NaCl, 500 mM imidazole, pH 7.4). The solution was then loaded on a Desalting column to get rid of imidazoles and excess salts.

### Effect on heat-induced aggregation

ADH from *Saccharomyces cerevisiae* and aldolase from rabbit muscle were used as substrate proteins to test the suppression effect of GreA on heat-induced aggregation. ADH bought from Sigma was diluted to 1 µM in 50 mM phosphate buffer (pH 7.4) and incubated at 48°C with different concentrations of GreA protein (0.2 µM, 0.5 µM, 1 µM, 2 µM). DnaK of 2 µM was also added as a control. The aggregation was monitored by detecting the optical density at 360 nm of the samples in an Ultrospec 2100 spectrophotometer (Amersham Biosciences).

Aldolase (GE Healthcare) was also diluted to 1 µM in 50 mM phosphate buffer (pH 7.4) and incubated at 50°C to induce aggregation. Various concentrations of GreA were added (0.5 µM, 1 µM, 2 µM), and aggregation was monitored as described above.

### Protection of enzymatic activity

ADH was diluted to 0.3 µM in 50 mM phosphate buffer (pH 7.4) with different concentrations of GreA (0.3 µM, 0.6 µM, 1.2 µM) or 1 µM DnaK added. Denaturation was induced by incubation in a 50°C water bath. After incubation for 80 min, the ADH activity was measured in reaction mixtures containing 50 mM phosphate buffer (pH 10.5), 5 mM NAD, and 5 mM ethanol. The reaction was started by adding ADH, and reduction of NAD was detected by the increase in absorbance at 360 nm.

### Reactivation of chemical denatured proteins

GFP was denatured at 100 µM in 0.12 M HCl for 60 min. Spontaneous refolding was initiated by 100-fold dilution in 50 mM Tris-HCl buffer (pH 7.5). Then, 3 µM GreA or 2 µM DnaK was added to the refolding system to test the effect. Refolding of GFP was monitored for 20 min by using a Fluostar Optima microplate reader with excitation at 400 nm and emission at 510 nm.

LDH (15 µM) was denatured in 6 M GnHCl at 25°C for 30 min. It was then diluted 100-fold (0.15 µM) into the refolding buffer (50 mM Tris-HCl, pH 7.2) in the presence of GreA at different concentrations (0.3 µM, 0.6 µM, or 1.2 µM) or 1 µM DnaK. After incubation at 25°C for 30 min, the LDH enzymatic activity was measured as described previously [35].

### Reactivation of heat-denatured proteins

LDH was diluted to 0.2 µM in 50 mM phosphate buffer (pH 7.4) and denaturation was started by incubation at 50°C for 80 min. After cooling to room temperature, different concentrations of GreA protein (0.2 µM, 0.4 µM, 0.8 µM) were added to start refolding, and the final concentration of LDH was adjusted to 0.1 µM. DnaK of 0.5 µM was also added as a control. The LDH activity was measured as described above.

### *In vitro* binding assays

Both SEC and non-denaturing gradient gel electrophoresis were used to detect the interaction between GreA and substrate proteins. LDH (15 µM) was denatured in 6 M GnHCl at 25°C for 30 min, and then diluted 50-fold (0.3 µM) into 50 mM Tris-HCl buffer (pH 7.2) in the presence or absence of GreA protein (1 µM). After incubation for 20 min at room temperature, the samples were filtered and subjected to SEC in a Superdex 200 HR column. Native LDH without denaturation was used as a control. The proteins were detected by their absorbance at 220 nm.

ADH (12 µM) was denatured in 6 M GnHCl at 25°C for 30 min and then diluted 40-fold (0.3 µM) with 50 mM Tris-HCl buffer (pH 7.2) in the presence or absence of GreA protein (4 µM). After incubation for 20 min at room temperature, loading buffer (bromophenol blue in 10% glycerol, 10 mM DTT) was added to the samples. Then the samples were loaded onto 5–12% non-denaturing gel (without SDS). The running buffer was 25 mM Tris-base/192 mM glycine.

### ANS binding assay

We used the hydrophobic probe ANS to determine the exposure of the hydrophobic domains by measuring the ANS fluorescence in a JASCO FP-6500 fluorescence spectrometer. The excitation wavelength was set at 370 nm, and the emission wavelength ranged between 400 nm and 600 nm. Purified GreA protein was incubated at 25°C, 45°C, or 50°C for 60 min and then mixed with 40 µM ANS to a final concentration of 2 µM. After incubation at 25°C for 20 min, the samples were scanned at a band pass of 3 nm.

### Circular dichroism (CD)

We used CD to detect the secondary structure stability of GreA. The purified GreA protein was diluted to 2 µM in 50 mM phosphate buffer and incubated at various temperatures (25°C, 45°C, 50°C) for 60 min. After they were cooled down, the samples were loaded onto a Jasco J-810 spectrometer in a cylindrical cell. Data were collected between 190 nm to 260 nm. We used the CDNN program to analyze the ratio of the secondary structures (kindly provided by Dr. Gerald Böhm, Institut für Biotechnologie, Martin-Luther Universität Halle-Wittenberg).

### Enhanced resistance of GreA-overexpressing strain

Both heat-shock resistance and oxidative resistance of the GreA-overexpressing strain were tested. The GreA-overexpressing strain and the control strain with the empty pET28a plasmid were cultured in LB medium to an OD_600_ of 0.4 and induced with 1 mM IPTG. After induction for 1 h, 10-µL bacterial liquids were diluted 1000-fold in pre-warmed 50 mM Tris-HCl buffer (pH 7.2) to 500 µL and then incubated at 48°C in water bath for 0 min, 20 min, 40 min, or 60 min. A 10-µL aliquot was then plated on LB agar plates and incubated at 37°C for 1 d. The viability of cells was estimated by counting the number of surviving cells on the plates.

To further examine the *in vivo* mechanism of GreA function, induced cells were harvested by centrifugation and washed once with 50 mM Tris-HCl buffer. Cells were resuspended in the same buffer and incubated at 48°C for 0 min or 40 min. The aggregated proteins in cells were isolated and detected, by using the modified method [36]. Bacterial liquid (5–50 mL) was cooled to 0°C on ice and centrifuged for 5 min at 5,000× *g* to harvest cells. Pellets were suspended in buffer A [10 mM phosphate buffer, pH 7.5, 1 mM EDTA, 20% sucrose (w/v), 1 mg/mL lysozyme] and incubated for 30 min on ice. After addition of buffer B [10 mM phosphate buffer, pH 7.5, 1 mM EDTA] (9-fold volume of buffer A), Cells were lysed by sonification. Intact cells were removed by centrifugation (2,000× *g*, 15 min). The insoluble fractions were isolated by centrifugation at 15,000× *g* (4°C, 20 min). Pellet was wash once and resuspended in 320 µL buffer B. Nonidet P-40 of 80 µL (10%, v/v) was added to remove the membrane proteins and the aggregates were isolated by centrifugation (15,000× *g*, 4°C, 20 min). This procedure was repeated. The insoluble aggregates were determined by Brandford assay.

The GreA-overexpressing and control strains were cultured and induced as above. Next, 10-µL aliquots of bacteria were diluted 1000-fold in 50 mM Tris-HCl buffer (pH 7.2) with 5 mM H_2_O_2_ to 500 µL. After incubation at room temperature for various time intervals, an aliquot of 10 µL was plated on LB agar plates and incubated at 37°C for 1 d. The viability of cells was estimated as mentioned above.

### *GreA/greB*-double mutation enhances cellular protein aggregation

The *greA/greB*-double mutant strain N6306 [29] and its control strain *E. coli* K12 MG 1655 were used to test the cellular protein aggregation. The control and N6306 strains were cultured in LB medium to an OD_600_ of 1.0 at 30°C. Cells were harvested and resuspended in 50 mM TrisHCl buffer. After heat shock at 48°C for 0 min or 40 min, the aggregates in cells were isolated and quantified as mentioned above.

To confirm the *in vivo* function of GreA, the *greA* gene was ligated to pET25b plasmid and transformed into N6306 strain. The N6306 strain with an empty pET25b plasmid was set as a control. Both the strains were cultured in LB medium to an OD_600_ of 1.0 at 30°C, and then plated on LB agar plates. The plates were incubated at 30°C or 42°C for 24 h. Both the strains were cultured at 30°C to an OD_600_ of 1.0, and then heat shocked as described above. The cellular aggregates are isolated and qualified the aggregates as above.
